# The Genotype (A to H) Dependent N-terminal Sequence of HBV Large Surface Protein Affects Viral Replication, Secretion and Infectivity

**DOI:** 10.3389/fmicb.2021.687785

**Published:** 2021-07-09

**Authors:** Guomin Ou, Lingyuan He, Luwei Wang, Ji Song, Xinyuan Lai, Xing Tian, Lei Wang, Kai Zhang, Xuechao Zhang, Juan Deng, Hui Zhuang, Kuanhui Xiang, Tong Li

**Affiliations:** ^1^Department of Microbiology and Infectious Disease Center, School of Basic Medical Sciences, Peking University Health Science Center, Beijing, China; ^2^Department of Clinical Laboratory, The Sixth Affiliated Hospital, Sun Yat-sen University, Guangzhou, China; ^3^Department of Clinical Laboratory Center, Beijing Children’s Hospital, Capital Medical University, National Center for Children’s Health, Beijing, China

**Keywords:** hepatitis B virus, HBV large surface protein, preS1 N-terminus, genotype, heterogeneity

## Abstract

Genetic variability has significant impacts on biological characteristics and pathogenicity of hepatitis B virus (HBV), in which the N-terminal sequence of the presurface 1 (preS1) region of HBV large surface protein (LHBs) displays genotype (GT) dependent genetic heterogeneity. However, the influence of this heterogeneity on its biological roles is largely unknown. By analyzing 6560 full-length genome sequences of GTA-GTH downloaded from HBVdb database, the preS1 N-terminal sequences were divided into four representative types, namely C-type (representative of GTA, GTB, and GTC), H-type (GTF and GTH), E-type (GTE and GTG), and D-type (GTD), respectively. We artificially substituted the preS1 N-termini of GTC and GTD plasmids or viral strains with each sequence of the four representative types. The roles of preS1 N-terminus on HBV replication, secretion and infectivity were investigated using HepG2 or HepG2-NTCP cells. In the transfection experiments, the results showed that the extracellular HBsAg levels and HBsAg secretion coefficients in D- and E-type strains were significantly higher than those in C- and H-type strains. D-type strain produced more extracellular HBV DNA than C-type strain. We further observed that D-, H-, and E-type strains increased the levels of intracellular replicative HBV DNAs, comparing with C-type strain. In the infection experiments, the levels of extracellular HBeAg, intracellular HBV total RNA and pgRNA/preC mRNA in D- and E-type strains were markedly higher than C and H-type ones. Our data suggest that the preS1 N-termini affect HBV replication, secretion and infectivity in a genotype dependent manner. The C- and H-type strains prefer to attenuate HBsAg secretion, while the strains of D- and E-type promoted infectivity. The existence and function of the intergenotypic shift of preS1 in naturally occurring recombination requires further investigation, as the data we acquired are mostly related to recombinant preS1 region between N-terminus of preS1 from genotypes A-H and the remaining preS1 portion of GTC or GTD.

## Introduction

Hepatitis B virus (HBV) causes more than 257 million chronic infections worldwide, and more than 880 thousand deaths each year from HBV-induced complications, such as hepatocellular carcinoma (HCC) (Revill et al., 2019; Howell, 2021). Although there are effective prophylactic vaccines and antiviral drugs, there has been so far no cure for chronic HBV infection. A better understanding of HBV biology is still urgently needed.

Hepatitis B virus is a prototype of the family Hepadnaviridae. There are two main types of HBV-related particles in patient sera, namely infectious Dane particles and non-infectious subviral particles (Hu and Liu, 2017). Both are enveloped by HBV surface protein (HBs), which is routinely measured in clinic as hepatitis B surface antigen (HBsAg) for the diagnosis and evaluating the treatment efficacy and prognosis (Cornberg et al., 2017; Liaw, 2019). HBs is encoded by presurface/surface (preS/S) gene, which transcribes 2.1 kb S mRNA under surface promoter II (SPII) or 2.4 kb preS mRNA under surface promoter I (SPI), respectively. The S mRNA translates into HBV small surface protein (SHBs) of 226 amino acid (aa) in length and HBV middle surface protein (MHBs) with additional 55 aa upstream (preS2 fragment) of the N-terminus of SHBs. The preS mRNA translates into HBV large surface protein (LHBs), which extends its N-terminal sequence (preS1 fragment) by different numbers of aa according to HBV genotype (GT) compared with MHBs.

This genotype-dependent length variation of preS1 fragment is the most striking difference among HBV genotypic sequences that have an overall >8% divergence among 10 genotypes (A-J) (Kramvis, 2014). There are 119 aa in GTA-GTC, GTF and GTH, 108 aa in GTD, and 118 aa in GTE and GTG, respectively. The length differences are concentrated in the aa 1–14 (numbering according to GTA-GTC) segment of preS1 rather than randomly occurring in the full-length sequence of preS1. Notably, LHBs is overlapped with HBV Polymerase (Pol) gene, which consists of terminal protein, spacer, reverse transcriptase, and RNaseH domains (Bartenschlager and Schaller, 1988; Radziwill et al., 1990). The spacer coding region corresponding to the preS1 N-terminus also display genetic heterogeneity. Although it is known that HBV genotypes vary in virological and clinical features in terms of HBV infection, replication, risk of HCC and response to antiviral therapy (Sunbul, 2014; Tong and Revill, 2016), the biological role of the divergent preS1 fragments is largely uncharacterized.

LHBs plays a critical role in viral morphogenesis and infection. Several studies have demonstrated that LHBs adopts two transmembrane topologies in the endoplasmic reticulum membrane (Loffler-Mary et al., 1997; Lambert, 2004). The cytosolic orientation of LHBs interacts with the nucleocapsid while the luminal orientation is required to localize the preS region on the surface of secreted virions to allow attachment to sodium taurocholate cotransporting polypeptide (NTCP) on the hepatocytes. Moreover, myristoylated preS1 (aa 2–48) is known to be essential for NTCP mediated HBV entry into hepatocytes (Schulze et al., 2010; Meier et al., 2013). Overproduction of LHBs by transgenic mice containing the preS/S open reading frame (ORF) has been shown to lead to the formation of extremely long subviral particles that are prone to accumulate within the ER of the hepatocytes and not efficiently secreted (Chisari et al., 1987). This is due to the retention property of LHBs, which is partially attributed to preS1 N-terminal sequence (Gallina et al., 1994).

The naturally occurring variants with preS1 N-terminal deletions have been extensively reported in patients with advanced disease progression, such as liver fibrosis and HCC (Lee et al., 2015; Choe et al., 2018; Li et al., 2021). We notice that many of these deletions may change 119 aa preS1 of GTA-GTC, GTF and GTH to 108 aa GTD-like preS1. It has been shown that the mutants with preS1 N-terminal substitutions among genotypes have distinct virological properties. The artificial deletion of preS1 N-terminal 11 aa in GTC showed comparable infectivity to GTD (Murayama et al., 2021). GTC strains with preS1 N-terminal deletion have been isolated from an advanced liver fibrosis patient and revealed to remarkedly enhance HBV replication and infectivity (Li et al., 2021); while the deletion of preS1 N-terminal 11 aa in GTA failed to observe this phenotype (Jiang et al., 2020). These studies reveal that heterogenous preS1 N-termini may confer HBV different infectivity and replication capacity *in vitro* according to viral genotype backgrounds. However, aforementioned studies were performed using plasmids carrying 1.1-mer HBV genomes under the control of foreign promoter, which hardly reflects the HBV life cycle under control of its own regulatory elements. Furthermore, the biological roles of preS1 N-terminal sequences of all known genotypes remain unclear.

In this study, for illustrating the biological roles of the genotype specific N-terminal sequences, we first constructed plasmids carrying 1.3-mer HBV genome of GTC or GTD, which is the predominant HBV genotype in Asia or Europe, respectively. We then substituted their preS1 N-terminal sequences with those of the well-known HBV genotypes (GTA-GTH) based on bioinformatic analysis. In total, 10 plasmids with GTC or GTD genetic background carrying various genotype-dependent preS1 N-terminal fragments were constructed and used for studying HBV replication and secretion in HepG2 cells. Additionally, the 1.05-mer counterparts of 1.3-mer HBV plasmids were also constructed. They were controlled by the cytomegalovirus (CMV) promoter, which benefited to the preparation of cell culture derived HBV variants for studying viral infectivity in HepG2-NTCP cells. The mutants’ LHBs were also expressed alone for investigating their impacts on HBV promoter activities by a dual luciferase reporter assay.

## Materials and Methods

### HBV Genome Sequences

The aligned full-length genome sequences of HBV GTA to GTH from HBVdb database available at https://hbvdb.lyon.inserm.fr/HBVdb/HBVdbIndex were downloaded. The sequences of GTA (*N* = 880), GTB (*N* = 1765), GTC (*N* = 2194), GTD (*N* = 1090), GTE (*N* = 306), GTF (*N* = 258), GTG (*N* = 40), and GTH (*N* = 27) were used to generate HBV genotype dependent consensus sequences (*N* = 8) by the function of Create Consensus Sequence in the BioEdit Sequence Alignment Editor Software (version 7.2.5, Ibis Therapeutics, Carlsbad, CA, United States) and their preS1 sequences were used for analysis by the same software.

### Plasmid Construction

The pBB4.5HBV1.2 plasmid carrying 1.2-mer genome length of HBV GTC used as a GTC template was reported in our previous study (Li et al., 2013). The 1.0-mer genome length of HBV GTD amplified from HepG2.2.15 cells, was cloned to pcDNA3.1 (Thermo Fisher Scientific, Waltham, MA, United States) (designated as 1.0D) and used as a GTD template in this study. The plasmids of HBV replication-competent wild type (*N* = 2) and variants (*N* = 8), for preparation of cell culture derived HBV (HBVcc) (*N* = 10), LHBs/MHBs/SHBs (LMS) expression (*N* = 1), LHBs expression (*N* = 4), and luciferase reporters of HBV promoters (*N* = 4) were constructed by the molecular cloning methods, such as polymerase chain reaction (PCR), overlap PCR, seamless cloning and site-directed mutation. The plasmid characteristics, the primer sequences and the detailed cloning strategies are summarized in the Supplementary Tables 1–3. In brief, the 1.3-mer HBV genomes of GTC (C) or GTD (D) was constructed by seamlessly cloning into pGEM3Z vector (Promega Corporation, Madison, WI, United States) from the relevant template plasmid, respectively. C-derived (CL1, C33, CH, and CE) or D-derived (DC, DCL1, DH, and DE) mutants were constructed by site-directed mutation and/or overlap PCR based on plasmid C or D. Plasmids (PC-C, PC-CL1, PC-C33, PC-CH, PC-CE, PC-D, PC-DCL1, PC-DC, PC-DH, and PC-DE) used for HBVcc production were generated by seamless clone, which carried 1.05-mer genome length from C- or D-derived strains under the control of CMV promoter. LMS coding region of C strain was cloned to pcDNA3.1 and LHBs expression plasmid (C-L) was generated by mutating start codon ATG of preS2 and S domain into codon CTG in LMS, while mutants’ LHBs (C33-L, CH-L, and CE-L) were constructed by PCR based on C-L. The HBV promoter sequences of core promoter (CP), SPI and SPII were cloned to pGL3-basic to control the expression of firefly luciferase. The CP were differentially designed as HBV pregenomic RNA (pgRNA) promoter (CP-pg) and precore (preC) mRNA promoter (CP-preC) according to a previous study (Moriyama, 1997) (Supplementary Figure 1). The pgRNA is the template for HBV DNA replication and encodes for HBV core protein (HBc) and Pol. The preC mRNA encodes for hepatitis B e antigen (HBeAg).

### Cell Culture and Transfection

HepG2 and HepG2-NTCP (gifts from Prof. Charles M. Rice, Rockefeller University, United States) were maintained in Dulbecco’s modified Eagle’s medium (DMEM) with 10% fetal bovine serum (FBS), 100 IU/ml penicillin, and 100 mg/ml streptomycin in collagen I-precoated plates in a 5% CO_2_ incubator at 37°C. HepG2 cells transfection was carried out using the Lipofectamine 2000 (Thermo Fisher Scientific) according to the manufacturer’s instruction.

### Production of HBVcc and HBV Infection

The 3 × 10^6^ HepG2 cells seeded in 10 cm dish were transfected with 18 μg plasmids containing 1.05-mer HBV genome under the control of CMV promoter. Three days post transfection, culture media were harvested and centrifugated at 5000 × *g* for 30 min to remove cell debris, followed by precipitation with 8% polyethylene glycol (PEG) 8000 at 4°C for overnight. The precipitates were resolved in DMEM and stored at −80°C. The HBVcc DNA was quantified by quantitative PCR (qPCR) using the 7500 Fast real-time PCR instrument (Thermo Fisher Scientific). The 5 × 10^4^ HepG2-NTCP cells were seeded in 24-well plate at day 1 and were infected by HBVcc at multiplicity of infection (MOI) of 5000 genome equivalents per cell (geq/cell) in DMEM with 3% FBS, 4% PEG8000, and 2% dimethyl sulfoxide (DMSO) at day 2. After 24 h infection, the cells were washed five times with PBS to remove residual HBV and HBsAg as much as possible, then cultured for 7 days in DMEM with 3% FBS and 2% DMSO. The culture media at 7 days post infection (dpi) were used for quantification of HBsAg and HBeAg by chemiluminescence immunoassay (CLIA) kits (Autobio diagnostics Co., Zhengzhou, China) and the cells were fixed for immunofluorescence assay.

### Western Blot

HepG2 cells (3 × 10^5^/well) in 6-well plate were transiently transfected with 2 μg plasmid C, D or their mutants. Three days after transfection, 0.5 ml culture supernatants were collected for immunoprecipitation by 2 μl horse anti-HBs antibody (Abcam, Cambridge, MA, United States) conjugated to 20 μl bed volume of Protein A/G PLUS-Agarose (Santa Cruz Biotechnology, California, United States) at 4°C overnight. The conjugated beads were lysed in 50 μl RIPA buffer (Beyotime, Shanghai, China), then stored at −80°C for use. The cells were lysed in 200 μl RIPA buffer with proteinase inhibitors on ice for 1 h. The proteins of HBs immunoprecipitation and cell lysate were loaded and separated via 12% SDS-PAGE, followed by transferring onto PVDF membranes (Merck KGaA, Darmstadt, Germany). Blotted membranes were blocked using 5% skim milk in TBST. Anti-HBs antibody allows to detect all three forms of HBs (S-, M-, and L- HBs) simultaneously, but the band intensity depends on the amount of each HBs. In this study, the mouse monoclonal anti-HBs antibody (Santa Cruz Biotechnology) was used to detect extracellular SHBs and the mouse monoclonal anti-preS1 antibody (Fitzgerald Industries International, Acton, MA, United States) was used for detection of extracellular LHBs. Intracellular HBs and HBc were detected using horse anti-HBs antibody (Abcam) or mouse monoclonal anti-HBc antibody (a gift from Prof. Guangxiang Luo, University of Alabama at Birmingham, United States), respectively.

### Extracellular HBV DNA Quantitative Analysis by Combining Immunoprecipitation With qPCR

HepG2 cells (5 × 10^4^/well in 24-well plate) were transfected with 500 ng plasmid C, D or their mutants. Three days after transfection, virions were immunoprecipitation from 0.3 ml of culture media by 1 μl horse anti-HBs antibody (Abcam) conjugated to 10 μl bed volume of Protein A/G PLUS-Agarose (Santa Cruz Biotechnology) at 4°C overnight. The immunoprecipitation was sequentially treated at 37°C with 2 μl Turbo DNase (Thermo Fisher Scientific) in manufacturer provided buffer for 1 h and 500 μl digestion buffer [25 mM Tris–HCl (pH 8.0), 0.5 mg/ml pronase (Merck KGaA), 0.5% SDS, 150 mM NaCl, and 10 mM EDTA] for 1 h, followed by DNA extraction with phenol. The extracted DNA was subjected to SYBR Green quantitative PCR using forward primer 5′-GACCACCAAATGCCCCTATC (2298–2317, numbering according to GTC) and reverse primer 5′-TGAGATCTTCTGCGACGCG (2412–2430, numbering according to GTC). The quantification range is 2.5 × 10^3^ to 2.5 × 10^11^ copies/ml.

### Northern Blot and Southern Blot

HepG2 cells (1.5 × 10^5^/well in 12-well or 3 × 10^5^/well in 6-well plate) were transfected with 1 or 2 μg plasmids. Three days after transfection, cells from 12-well plate were harvested for total RNA extraction by Trizol Reagent (Thermo Fisher Scientific). After isopropanol precipitation and 75% ethanol wash, RNA pellets were resolved in 40 μl RNase-free water, then quantified by NanoDrop (Thermo Fisher Scientific). After heat denaturation, 20 μg of total RNA was separated in a 1.5% agarose gel containing MOPS and formaldehyde. RNAs were blotted onto Nylon Membranes (positively charged) (Roche Diagnostics, Basel, Switzerland) and hybridized with Dig-labeled HBV probe (3.2 kb). The probe was generated using PCR with forward primer 5′-CTAATCATCTCATGTTCA, reverse primer 5′-GCAGAGGTGAAAAAGTTGCATGGTGCTG and digoxigenin-11-dUTP (Roche Diagnostics). HepG2 cells from 6-well plate were lysed in 500 μl lysis buffer [10 mM Tris–HCl (pH 8.0), 1 mM EDTA, 1% NP-40, and 2% sucrose]. The HBV core particles were precipitated by adding 120 μl 35% PEG8000 containing 1.5 M NaCl to the cell lysate, followed by Turbo DNase digestion, pronase digestion, phenol extraction, and DNA precipitation. The 20 μg extracted DNA was separated in a 1.2% agarose gel, and transferred to Nylon Membranes (positively charged) using 20 × SSC solution. Southern blots were also hybridized with Dig-labeled HBV probe as mentioned above, followed by Anti-Digoxigenin-AP Fab fragments (Roche Diagnostics) incubation for 30 min at room temperature and exposure in ChemiDoc XRS Imaging System (Bio-Rad, Hercules, California, United States).

### Intracellular HBV RNA Detection by Reverse Transcription-Quantitative PCR (RT-qPCR)

Total RNA was extracted from cultured cells using Trizol reagent according to the aforementioned description. RNA samples were reverse transcribed by RevertAid First Strand cDNA Synthesis Kit (Thermo Fisher Scientific) according to manufacturer’s instruction. The cDNA was subjected to SYBR Green quantitative PCR. The primers for pgRNA/preC mRNA detection are the same to those of extracellular HBV DNA quantification. HBV total RNA was quantified using forward primer 5′-GCACCAGCACCATGCAAC (1803–1820, numbering according to GTC) and reverse primer 5′-AAGCCACCCAAGGCACAG (1877–1984, numbering according to GTC). The endogenous control (ribosomal protein S11 gene) was quantified using forward primer 5′-GCCGAGAC TATCTGCACTAC and reverse primer 5′-ATGTCCAGCC TCAGAACTTC. 2^–ΔΔCT^ quantitative method was applied to calculate HBV RNA relative expression levels.

### Dual Luciferase Reporter Assay

HepG2 cells (5 × 10^4^/well in 24-well plate) were co-transfected with 500 ng HBV promoter plasmid (CP-pg, CP-preC, SPI, or SPII) and 5 ng plasmid pRL-TK (Promega Corporation) carrying renilla luciferase for normalizing in the absence and presence of 500 ng LHBs expression plasmid (C-L, C33-L, CH-L, or CE-L). Three days after transfection, HBV promoter activities were determined by measuring luciferase activity using the Dual Luciferase Reporter Assay System (Promega Corporation).

### Chemiluminescence Immunoassay

The HBsAg (quantification range: 0.05–250 IU/ml) and HBeAg (quantification range: 0.1–200 PEI U/ml) were quantified by CLIA kits (Autobio diagnostics Co., Zhengzhou, China) according to the manufacturer’s instructions.

### Immunofluorescence and Confocal Microscopy

HepG2-NTCP cells were cultured in 24-well plate for HBV infection as aforementioned. The 1 × 10^5^ HepG2 cells were seeded onto collagen I-precoated glass coverslips in 12-well plates and then transfected with 1 μg C-derived or D-derived plasmids for three days. After infection or transfection, cells were fixed in 4% paraformaldehyde (PFA) for 10 min, washed twice with PBS, and permeabilized using 0.1% Triton X-100 in PBS for 10 min at room temperature. Then, cells were blocked with 10% goat serum and 1% BSA in PBS for 1 h at room temperature. The cells were then incubated with primary antibody at 4°C overnight. After 3 times washing in PBS with 0.1% Tween 20, fluorescence-labeled secondary antibodies and DAPI using for nuclear staining were subjected to the cells at room temperature for 1 h. The final concentrations of primary antibodies were 1:1000 for rabbit polyclonal anti-HBc antibody (Fitzgerald Industries International) and 1:300 for mouse monoclonal anti-preS1 antibody (Santa Cruz Biotechnology). The secondary antibodies conjugated with Alexa Fluor 594 (goat anti-rabbit IgG; Thermo Fisher Scientific) or Alexa Fluor 488 (goat anti-mouse IgG; Thermo Fisher Scientific) were used at 1:1000 dilution. The samples were imaged with a fluorescence microscope (RVL-100-G, ECHO) or a confocal laser scanning microscope (TCS-SP8, Leica).

### Statistical Analysis

All experiments were repeated at least three times in an independent manner and three replicates were performed each time. All data were plotted using Origin 2019 software (OriginLab Corporation, Northampton, MA, United States) and statistically analyzed using SPSS Statistics 19 software (IBM Corporation, Armonk, NY, United States) and showed as the means ± SD. Statistical significance was tested using a two-tailed *t* test for two-sample comparisons. The *p* value less than 0.05 was considered as statistically significant.

## Results

### Genotype Dependent Heterogeneity of HBV preS1 N-terminal Sequences

**FIGURE 1:**
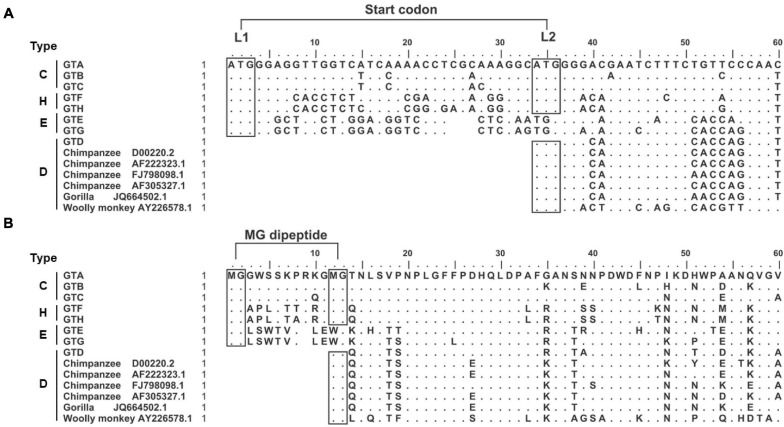
Genotype dependent heterogeneity of HBV preS1 N-terminal sequences from primate hepadnaviruses. **(A)** Alignment of 5′ preS1 nucleotide sequences. The GTA-GTH sequences are consensuses from alignments in HBVdb database. The numbering and sequence alignment are based on GTA. Dot means homologous to GTA, while space indicates deletion. The boxes with “ATG” indicate (potential) start codon, which is named as L1 and L2, respectively. **(B)** Alignment of preS1 N-terminal aa sequences. The boxes with “MG” indicate (potential) myristoylation motif. The preS1 N-terminal sequences were divided into four types. C-type represents preS1 (aa 1–14) in GTA-GTC; H-type represents preS1 (aa 1–14) in GTF and GTH; E-type represents preS1 (aa 1–13) in GTE and GTG; D-type represents preS1 (aa 1–3) in GTD and hepadnaviruses from non-human primates. aa, amino acid; GT, genotype; nt, nucleotide.

Our study strategy was to replace or put the genotype specific preS1 N-terminal sequences in the GTC or GTD sequence backbones and to illustrate their biological function under the consistent genetic background. First, 6560 HBV genomic sequences including GTA-GTH alignments were downloaded from HBVdb database, then the genotype specific consensus sequences were generated by BioEdit Sequence Alignment Editor. Their 5′ preS1 nucleotide and preS1 N-terminal aa consensus sequences and 6 genome sequences from non-human primate (Chimpanzee, Gorilla and Wooly monkey) hepadnaviruses were aligned (Figures 1A,B). Based on the length and sequence variations of the preS1 N-termini, we found that they could be classified into 4 types (C, H, E, and D). As an example, C-type was a representative of the preS1 aa 1–14 in GTA-GTC. So were H-type (representative of GTF and GTH), E-type (GTE and GTG), and D-type (GTD and animal hepadnaviruses).

Second, the sequence features important for clone design or data interpretation were analyzed as follows. We noticed that the preS1 N-termini of C- and H-types harbored two in-frame start codons (designated as L1 and L2), while E- and D-types only had one start codon corresponding to L1 and L2, respectively (Figure 1A). In addition, N-terminal MG dipeptide is a conserved myristoylated motif in LHBs and the myristoylation was reported to be important for viral infectivity (Schulze et al., 2010; Meier et al., 2013). We found that the preS1 N-termini of C- and H-types harbored two MG motif within aa 1–13 but E- and D-types only had one such motif (Figure 1B). These brought the possibility that the loss of L1 as seen in some clinical samples (Choe et al., 2018; Li et al., 2021) may allow LHBs to begin translation from L2, to produce D-type LHBs with potential myristoylation ensuring infectivity. Moreover, hydrophobicity and hydrophilicity profiles of preS1 N-terminal aa sequences were predicted by BioEdit Sequence Alignment Editor (Supplementary Figure 2). The results showed that N-termini of preS1 could be divided as four types as their sequences, and C- and H-types showed similar profiles of hydrophobicity and hydrophilicity.

### PreS1 N-terminal Sequences Affect HBV Proteins Expression and Secretion

**FIGURE 2:**
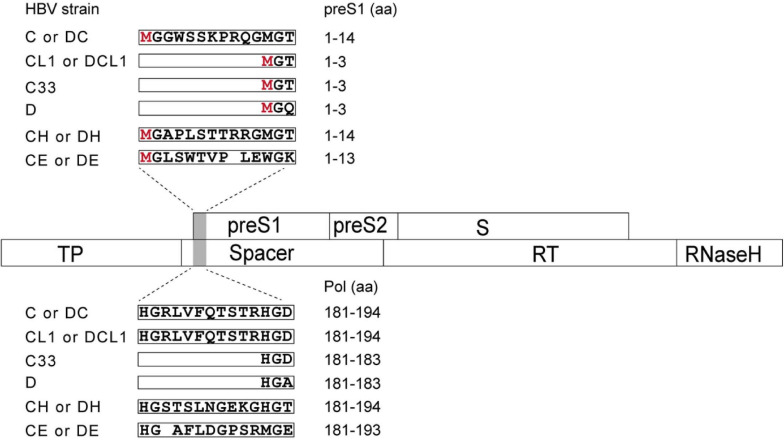
Schematic representation of preS1 N-terminal sequences from C strain (GTC) or D strain (GTD) and their mutants in this study. Overlapping feature is showed between preS/S and Pol ORFs, which are indicated by open boxes. Mutated region is shaded by gray color. N-terminal sequences of preS1 are translated on the top in open boxes and corresponding Pol-spacer sequence are translated below. CL1 represents the C strain with mutated L1 from ATG into ACG, which shortens N-terminus of preS1 but changes no aa in spacer. C33 represents C with 33 nt deletion corresponding to 11 aa deletion at N terminus of preS1, which deletes 11 aa in both preS1 and spacer. CH and CE represent C with preS1 N-terminus substitution with H- and E-type, respectively. DC, DH and DE represent D with preS1 N-terminal insertion of C-, H- and E-type, respectively. DCL1 represents DC with mutated L1 from ATG into ACG, which recoveries preS1 N-terminus to D-type but extends spacer by 11 aa to make it as C-type. HBV, hepatitis B virus; preS, presurface; S, surface; TP, terminal protein; RT, reverse transcriptase; ORF, open reading frame; aa, amino acid.

To study the impact of N-terminal sequences from GTA-GTH on HBV life cycle, the following mutants were generated. First, the C strain (GTC) was used as a genetic backbone to make HBV replication-competent mutants (Figure 2 and Supplementary Table 1). The CL1 represents C strain with mutated L1 from ATG into ACG, which shortens N-terminus of preS1 but does not change aa sequence of Pol-spacer. C33 represents C strain with 33 nt deletion at 5′ preS1 nucleotide sequences, which results in 11 aa deletion in both preS1 and Pol-spacer. CH and CE represent C strain with preS1 N-terminal replacement of H-type or E-type, respectively. Second, the D strain (GTD) as a genetic backbone was used to construct the HBV mutants in a similar way as for C but using insertion instead of deletion strategies. The DC, DH, and DE strain represent D strain with preS1 N-terminal sequence insertion from the C-, H-, or E-type sequence, respectively (Figure 2 and Supplementary Table 1). DCL1 represents DC with mutated L1 from ATG into ACG, which recoveries preS1 N-terminus to D-type but keeps Pol-spacer as that in DC (Figure 2).

**FIGURE 3:**
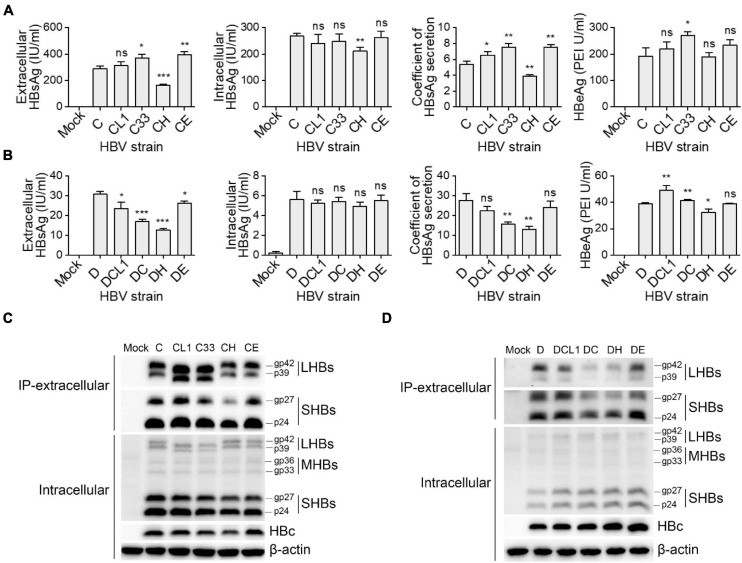
Impact of preS1 N-terminal sequences on HBV protein expression and secretion. Plasmids containing 1.3-mer HBV genome from C-derived **(A,C)** and D-derived **(B,D)** strains were transfected into HepG2 cells. Cells and culture supernatant were harvested at day 3 post transfection. **(A,B)** Extracellular HBsAg and HBeAg and intracellular HBsAg were determined by CLIA kit. **(C,D)** Western blot detection of intracellular HBs (LHBs, MHBs, and SHBs) and HBc. Extracellular SHBs or LHBs were also detected by Western blot after HBs immunoprecipitation with anti-HBs antibody (Abcam). The glycosylated (gp) and non-glycosylated (p) forms of HBs are indicated. Histograms of **(A,B)** show mean values from one representative experiment. Bars indicate Standard Deviation. The *p* values were determined using Student’s *t* test; ns represents no significant, *p < 0.05, **p < 0.01, ***p < 0.001. CLIA, chemiluminescence immunoassay; IP, immunoprecipitation; HBV, hepatitis B virus; HBsAg, hepatitis B surface antigen; HBeAg, hepatitis B e antigen; LHBs, HBV large surface protein; MHBs, HBV middle surface protein; SHBs, HBV small surface protein; HBc, HBV core protein.

These 10 plasmids were transfected into HepG2 cells, respectively, then the expression and secretion of HBV proteins were assayed after 3 days. The GTC-derived mutants were compared to the C wild type strain, the results in Figure 3A showed that C33 and CE increased extracellular HBsAg levels and HBsAg secretion coefficients, while CH significantly decreased these two parameters (secretion coefficient of C/C33/CE/CH: 5.4/7.5/7.6/3.9, *p* < 0.05). For GTD-derived strains, DC and DH lowered the secretion coefficients of HBsAg but DE showed no significant change of it (secretion coefficient of D/DC/DH/DE: 27.7/15.7/13.0/24.1, *p* < 0.05) (Figure 3B). Further dissection of HBsAg by Western blot revealed that LHBs of CL1 and C33 showed lower molecular weight (MW) than that of C strain as expected. Intriguingly, CL1 and C33 markedly elevated the levels of extracellular LHBs but not SHBs (Figure 3C). DC and DH significantly reduced the amount of extracellular LHBs and SHBs compared with those of D wild type (Figure 3D). For intracellular SHBs, the mutants of C strain seemed to have less band intensities (gp27 and gp24) than those of C strain, while the mutants of D strain showed opposite (Figures 3C,D). In addition, CH produced the weakest HBc band signal among all C-derived strains, while DH and DE showed stronger HBc signal than the other D-derived strains (Figures 3C,D).

### PreS1 N-terminal Sequences Affect the Subcellular Distribution of LHBs

The effect of preS1 N-terminal sequences on the subcellular distribution of LHBs was analyzed by confocal immunofluorescence microscopy. As showed in Supplementary Figure 4A, LHBs was found to be located in the cytoplasm with a spotty aggregation distribution in C and CH strains, but a dispersed distribution in CL1, C33, and CE strains. DC and DH strains also exhibited obvious spotty aggregation distribution as seen in C and CH strains (Supplementary Figure 4B), indicating the preS1 N-terminal sequence from C-type or H-type is able to induce LHBs to aggregate in the cytoplasm regardless of its overall GTC or GTD genetic background.

### Impact of preS1 N-terminal Sequences on Extracellular HBV DNA Levels

**FIGURE 4:**
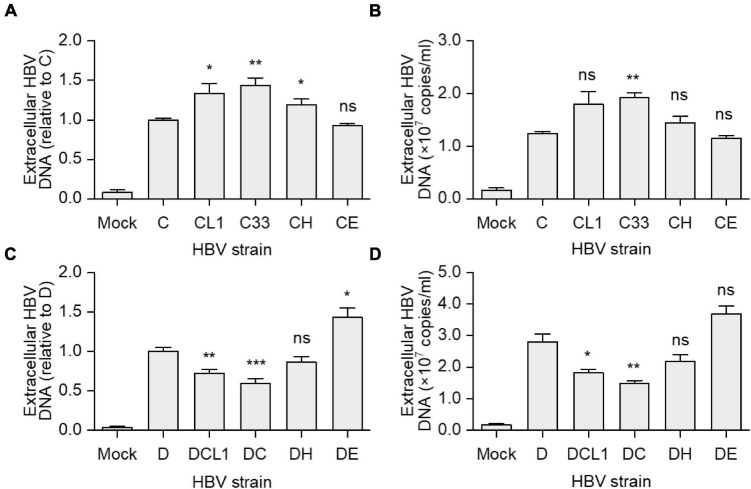
Impact of preS1 N-terminal sequences on extracellular HBV DNA levels. HepG2 cells were transfected with plasmids containing 1.3-mer HBV genomes from C-derived **(A,B)** or D-derived **(C,D)** strains. **(A,C)** Data represent means ± SEM from two independent experiments, and the value for C or D strain were normalized as 1.0. **(B,D)** The histograms show mean values from one representative experiment; the bars indicate Standard Deviation. The *p* values were determined using Student’s *t* test; ns represents no significant, *p < 0.05, **p < 0.01, ***p < 0.001. HBV, hepatitis B virus.

Hepatitis B virus virions were immunoprecipitation from culture supernatant of HepG2 cells transfected with strain C, D, and their mutants. The virion-associated HBV DNA was extracted and detected by qPCR. CL1 and C33 strains had higher extracellular HBV DNA levels than C strain [HBV DNA level of C/CL1/C33 (copies/ml): 1.2 × 10^7^/1.8 × 10^7^/1.9 × 10^7^, *p* < 0.05], while CH and CE barely changed it (Figures 4A,B). Conversely, DCL1 and DC showed significantly lower HBV DNA levels compared with D [HBV DNA level of D/DCL1/DC (copies/ml): 2.8 × 10^7^/1.8 × 10^7^/1.5 × 10^7^, *p* < 0.05] (Figures 4C,D). The results suggest that the change of a short fragment of preS1 N-terminal of LHBs might affect the extracellular virion amounts.

### Impact of preS1 N-terminal Sequences on HBV Transcription and Replication

**FIGURE 5:**
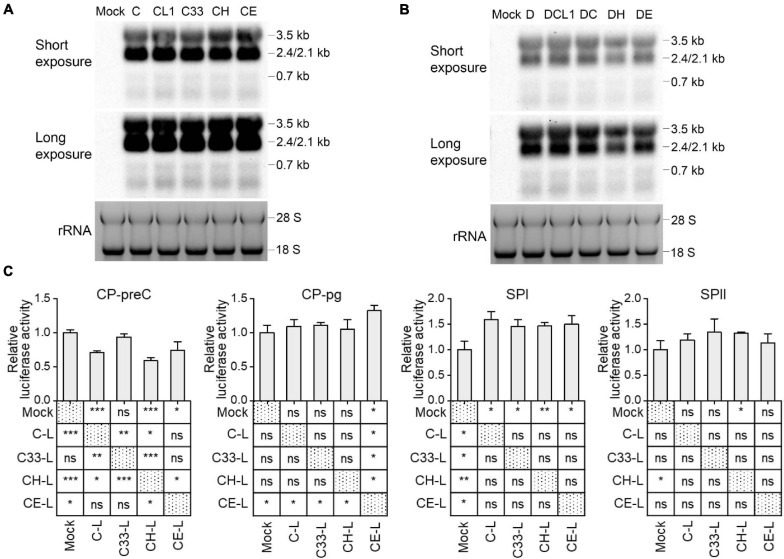
Impact of preS1 N-terminal sequences on HBV transcription. Northern blot of intracellular HBV RNAs from HepG2 cells transfected with 1.3-mer HBV genome from C-derived **(A)** or D-derived **(B)** strains, with ethidium bromide stained rRNA serving as a loading control. **(C)** HepG2 cells were transiently co-transfected with HBV promoter plasmid (CP-pg, CP-preC, SPI, or SPII), which controls the expression of firefly luciferase, LHBs expression plasmid (C-L, C33-L, CH-L, or CE-L) and normalizing plasmid pRL-TK. Three days after transfection, the promoter activities were determined by dual luciferase reporter assay. Relative luciferase activity was calculated as firefly/renilla rate and further normalized as fold change to Mock. **(C)** The histograms show mean values from one representative experiment; the bars indicate Standard Deviation. The *p* values were determined using Student’s *t* test; ns represents no significant, *p < 0.05, **p < 0.01, ***p < 0.001. CP, core promoter; SPI, surface promoter I; SPII, surface promoter II; rRNA, ribosomal RNA.

Although the preS1 N-terminal sequence has no overlap with HBV promoter sequences, LHBs has been reported to have transcription activation function (Hildt et al., 1996; Ryu et al., 1997). To test whether mutated LHBs would affect HBV transcription, Northern blot was applied. Each of HBV RNAs (3.5, 2.4/2.1, and 0.7 kb) displayed similar levels among C-derived and D-derived strains, respectively (Figures 5A,B). However, this assay allows only to observe marked differences. RT-qPCR detection showed that the levels of HBV total RNA and pgRNA/preC mRNA had no significant difference among C-derived or D-derived strains (Supplementary Figure 5). Since HBV transcripts are still difficult to be exactly distinguished and their levels might be affected by transcription, HBV DNA replication and viral translation, no obvious differences in Northern blot and RT-qPCR does not necessarily mean no difference in transcriptional efficacies among mutants. Therefore, the influence of mutated LHBs on HBV promoter activity was further studied using various LHBs from C-derived mutants and dual luciferase reporter assay.

Plasmids expressing C and its mutants-derived LHBs were constructed using pcDNA3.1 vector and transiently transfected to HepG2 cells (Supplementary Table 3). Western blot showed that the band intensities of intracellular LHBs from C33 (named C33-L) and CE (CE-L) were significantly weaker than those from C (C-L) and CH (CH-L) (Supplementary Figure 3). In line with the previous findings (Chisari et al., 1986; Ou and Rutter, 1987), extracellular LHBs was not detected (data not shown). The introduction of various LHBs into HBV reporter systems (Supplementary Figure 1) showed that the most affected promoters were CP-preC and SPI, but CP-pg and SPII were less affected (Figure 5C and Supplementary Table 4). The CP-preC activity was significantly reduced by C-L (*p* < 0.001), CH-L (*p* < 0.001), and CE-L (*p* < 0.05) compared with the mock and the degree of influence depended on genotype. In contrast, the SPI activity was significantly increased by all mutants’ LHBs (*p* < 0.05) with no genotypic difference.

Furthermore, HBV replicative DNAs containing relaxed circular (RC), double-stranded linear (DSL) and single stranded (SS) DNAs were detected post transfection by Southern blot. C33, CH, and CE produced stronger signal than C and CL1 (Supplementary Figure 6A). DC showed the weakest signal among all D-derived strains (Supplementary Figure 6B).

### Impact of preS1 N-terminal Sequences on HBV Infectivity in HepG2-NTCP Cells

**FIGURE 6:**
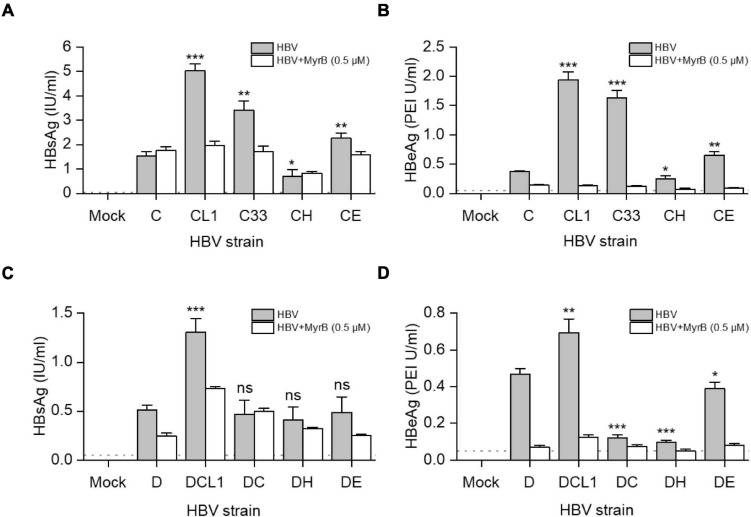
Impact of preS1 N-terminal sequences on HBV infectivity in HepG2-NTCP cells. HepG2 cells were transfected with plasmids containing 1.05-mer HBV genome from C-derived **(A,B)** or D-derived **(C,D)** strains under the control of CMV promoter. Three days post transfection, culture supernatants were collected and concentrated for HBV virion preparation. The multiplicity of infection of 5000 genome equivalents per cell were inoculated into HepG2-NTCP cells. MyrB was used for blocking HBV entry. Extracellular HBsAg **(A,C)** and HBeAg **(B,D)** were determined by CLIA at day 7 post infection. The dashed lines indicate the lower limit of quantification of HBsAg (0.05 IU/ml) or HBeAg (0.1 PEI U/ml). The histograms show mean values from one representative experiment. The bars indicate Standard Deviation. The *p* values were determined using Student’s *t* test; ns represents no significant, *p < 0.05, **p < 0.01, ***p < 0.001. HBV, hepatitis B virus; MyrB, Myrcludex B.

HBVcc were generated from HepG2 cells transfected with 1.05-mer HBV genome length of C, D and their mutants under the control of CMV promoter. Culture supernatant was harvested 3 days post transfection and HBV was pelleted by PEG8000, then HBV DNA titers were measured by qPCR. We inoculated HBVcc to HepG2-NTCP cells with equal MOI and cultured for 7 days. As showed in Figures 6A,B, the extracellular HBsAg [C/CL1/C33/CE: 1.5/5.0/3.4/2.3 (IU/ml), *p* < 0.05] and HBeAg [C/CL1/C33/CE: 0.4/1.9/1.6/0.7 (PEI U/ml), *p* < 0.05] levels were significantly higher after CL1, C33, and CE infection than those of C infection. The blockage of infection by 0.5 μM Myrcludex B (MyrB) and presence of detectable HBeAg ensured what we measured were highly likely from the *de novo* infection; and HBeAg should be a better parameter than HBsAg to evaluate HBV infectivity due to no potential residual HBeAg in the inocula. Thus, although only DCL1 had the highest extracellular HBsAg level among D-derived strains (Figure 6C), we believed that D, DCL1, and DE had significantly higher infectivity than that of DC and DH, respectively, based on their HBeAg [D/DCL1/DE/DC/DH: 0.5/0.7/0.4/0.1/0.1 (PEI U/ml), *p* < 0.05] levels (Figure 6D).

Furthermore, the infectivity of HBV from C, D and their mutants was analyzed by immunofluorescence assay. There were more HBc-positive cells post CL1 and C33 infection than other C-derived strains, although the efficiency of infection from HBVcc was low (Supplementary Figure 7A). The introduction of preS1 N-terminal sequence from type C or H to D strain markedly reduced the number of HBc-positive cells, while DCL1 mutating first start codon of preS1 N-terminus from DC recovered its infectivity. The E-type sequence fused to preS1 N-terminus of D strain also preserved HBV infectivity (Supplementary Figure 7B).

In addition to detection of HBV protein levels, the intracellular HBV RNAs were quantified by RT-qPCR post infection. CL1, C33, and CE transcribed more HBV total RNA [CL1/C33/CE/CH (relative to C): 2.8/3.0/1.7/0.7, *p* < 0.05] and pgRNA/preC mRNA [CL1/C33/CE/CH (relative to C): 2.9/3.2/1.7/0.8, *p* < 0.05] than those of C and CH; similarly, D, DCL1 and DE showed higher levels of total RNA [DCL1/DE/DC/DH (relative to D): 0.5/0.4/0.05/0.02, *p* < 0.05] and pgRNA/preC mRNA [DCL1/DE/DC/DH (relative to D): 0.4/0.5/0.05/0.02, *p* < 0.05] than those of DC and DH (Supplementary Figure 8).

## Discussion

Large surface protein consists of preS1, preS2, and S domains and plays pivotal roles in morphogenesis of HBV and infection of hepatocytes. Among these domains, the preS1 is the most mutation-prone, especially at its mystery N-terminus (Lee et al., 2015; Choe et al., 2018; Li et al., 2021). Evolution makes it with different sequence and length depending on HBV genotypes. However, the biological roles of this N-terminal sequence from different HBV genotypes are still unclear. In this study, we constructed HBV mutants in the GTC and GTD genetic backbones with modified preS1 N-termini mimicking those in the LHBs of GTA-GTH. The characteristics of the mutants are summarized in Supplementary Table 5, which indicates that the heterogenous preS1 N-terminus sequence might affect the replication, secretion and infection of HBV through transcription regulation, cellular retention, secretion and infectivity change in a genotype-dependent manner.

In this study, GTC and GTD were chosen as the genetic backbones for constructing the mutants due to their higher prevalence in Asia and Europe (Sunbul, 2014), and their ever use in many *in vitro* studies including ours (Xiang et al., 2017). But different from previous studies, the HBV replication of our plasmids harboring 1.3-mer HBV genome is driven by HBV CP rather than the foreign promoters, such as CMV promoter (Jiang et al., 2020; Li et al., 2021). Our plasmids may better reflect the nature HBV replication that is important for a study trying to compare up to 10 HBV strains. In addition, we notice that the intergenotypic shift of preS1 in naturally occurring recombination might exist according to some previous reports (Simmonds and Midgley, 2005; Abdou Chekaraou et al., 2010), it would be interesting for further investigation in patients.

To analyze the biological roles of genotype specific preS1 N-termini, we begin with the C33 and DC that changed their LHBs preS1 N-terminus and Pol-spacer into each other’s genotype. Interestingly, this exchange alters the level of many viral markers toward each other (Supplementary Table 5). For instance, D-type of C33 strain increased extracellular HBsAg, LHBs and HBV DNA levels and infectivity to HepG2-NTCP cells, while C-type of DC strain decreased them, respectively. Our results suggest that the LHBs harboring D-type preS1 N-terminus has less cellular retention effect than that of C-type (Chisari et al., 1986; Ou and Rutter, 1987). It also confers stronger infectivity as previously reported (Li et al., 2021; Murayama et al., 2021). Further analysis on CH and DH strains shows significant decline of extracellular HBsAg, LHBs and SHBs levels, and infectivity regardless of their genetic backbones. This is the first report suggesting that the H-type (including GTF and GTH) preS1 N-terminal sequence probably confers the strongest secretion inhibition capacity on HBsAg (Figures 3C,D). For E-type, its promotion on HBsAg secretion and infectivity is seen in CE strain but not in DE strain, which indicated that preS1 N-terminus could have a different behavior according to the genetic backbones of the different HBV genotypes. However, when DE strain is compared with DC and DH, it always shows better HBsAg secretion capacity and higher infectivity.

According to these analyses, we suggest that the capacity of the preS1 N-terminus of LHBs to retain HBsAg in the cells is probably as follows: H-type > C-type > E-type > D-type; while its effect on infectivity is in reverse order. To our knowledge, this ranking has not been reported in previous studies. In addition, although the extracellular HBV DNA levels were different among the mutants in the transfection experiments (Figure 4), the same MOI was used in the infection assay to avoid variations of virion amounts in the inocula. Thus, we think it is reasonable to speculate that the genotype specific role of preS1 N-terminus on infectivity is largely attributed to the sequence variations *per se*.

It is known that overexpression of LHBs abolishes HBsAg secretion *in vivo* (Chisari et al., 1986) and *in vitro* (Ou and Rutter, 1987). In line with these, the intracellular LHBs level in those with strong inhibition of HBsAg secretion (C-type and H-type) was higher than those with weak inhibition (D-type and E-type). This could be found in the results of 1.3-mer HBV plasmid transfection and LHBs expression experiments (Figures 3C,D and Supplementary Figure 3). Moreover, LHBs of C-type and H-type displayed spotty aggregation distribution while D-type and E-type dispersedly distributed in the cytoplasm, suggesting the modification of LHBs secretion pathway. This observation is in agreement with a previous finding that fusion of the GTA-derived preS1 N-terminal 11aa (corresponding to C-type in our study) to eGFP caused significant subcellular aggregation of eGFP, while GTE-derived N-terminus fusion protein showed dispersed distribution (Jiang et al., 2020).

Regarding HBV infectivity, the present study shows that D-type and E-type LHBs enhanced HBV infectivity compared with C-type and H-type in both GTC and GTD backgrounds. A previous study also shows that the LMS of GTD strongly supported the infection of hepatitis D virus (Freitas et al., 2014). This might partially due to a stronger capacity of binding to NTCP by preS1 domain of GTD than that of GTC (Li et al., 2021). Of note, those (D-type and E-type) conferring better infectivity are the ones with one start codon, one myristoylation motif, and similar hydrophilicity in their preS1 N-terminal sequences. Myristoylation of preS1 N-terminus is necessary for HBV infection (Bruss et al., 1996; Schulze et al., 2010; Meier et al., 2013). It is also known that the occurring and the degree of myristoylation is not only dependent on the N-terminal MG motif but also its near-by downstream sequence (Utsumi et al., 2004). Based on sequence analysis, the preS1 N-termini of GTD-GTH prefer myristoylation, while those of GTA-GTC are prone to direct heterogeneous modification with both myristoylation and acetylation (Utsumi et al., 2004). This may account for the higher infectivity in D-type and E-type than C-type, however, this does not seem to be the explanation for the lower infectivity in H-type. Although we do not know if this might also be due to the difference of the hydrophobicity and hydrophilicity of preS1 N-termini (Supplementary Figure 2), we do suggest more studies to understand the underlying mechanisms.

It is known that LHBs has transactivation activity (Hildt et al., 1996; Ryu et al., 1997). In an early study, Kim et al. (Ryu et al., 1997) compared the transactivation capacities of preS1 (119 aa, adr), preS1 (119 aa, adw), and preS1 (108 aa, ayw) domains fused to the DNA-binding domain of transcriptional activator GAL4, which would activate the promoter of the GAL1. The 108 aa preS1 corresponding to our D-type preS1 N-termini (C33-L) was found to display the highest transactivation activity. Instead, we studied this by testing the activities of luciferase reporters of HBV own promoters in the absence and presence of full-length LHBs. We found that various LHBs had different influence on CP-preC, CP-pg, SPI, and SPII transcription activities, of which CP-pg and SPII were less affected and SPI was exclusively up-regulated by all tested LHBs. The C-type (C-L), H-type (CH-L), and E-type (CE-L) LHBs significantly inhibited transcription of preC mRNA (Figure 5C). In addition, D-type LHBs barely affected the activities of CP-preC, CP-pg, and SPII. Our data revealed that the identical LHBs differentially regulated the activities of different HBV promoters, and LHBs carrying different preS1 N-termini also divergently regulated the activity of the identical HBV promoter.

In conclusion, our data reveal the functions of preS1 N-termini in HBV life cycle. The N-terminal sequences of preS1 not only regulate the secretion of HBsAg but also alter the replication and infectivity of HBV in a genotype specific manner. The existence and function of the intergenotypic shift of preS1 in naturally occurring recombination requires further investigation, as the data we acquired are mostly related to recombinant preS1 region between N-terminus of preS1 from genotypes A-H and the remaining preS1 portion of GTC or GTD. From the practical point of view, our findings also indicate a necessity to consider the influence of HBV genotypes in antiviral drug screening, especially when screening for entry inhibitors.

## Data Availability Statement

The raw data supporting the conclusions of this article will be made available by the authors, without undue reservation.

## Author Contributions

GO, TL, KX, and HZ conceived and designed the research. TL, KX, and HZ contributed reagents, materials, supervised work, and gave critical review on and revised the manuscript. GO, LH, LuW, JS, XL, XT, LeW, KZ, XZ, and JD carried out the experiments. GO performed the statistical analyses and wrote the first draft of the manuscript. All authors contributed to the article and approved the submitted version.

## Conflict of Interest

The authors declare that the research was conducted in the absence of any commercial or financial relationships that could be construed as a potential conflict of interest.
