# Supplementary material for: The Genotype (A to H) Dependent N-terminal Sequence of HBV Large Surface Protein Affects Viral Replication, Secretion and Infectivity
Source: Front Microbiol. 2021 Jul 9;12:687785. doi: 10.3389/fmicb.2021.687785 (PMC8299529; doi:10.3389/fmicb.2021.687785)
Supplement: Supplementary file 1 [file Data_Sheet_1.docx]

**Supplementary Table S1. HBV strains and their characteristics**

| **Strain** | **Mutation in LHBs** | **LHBs-N-terminus** | **Pol-spacer** |
| --- | --- | --- | --- |
| C | No, defined as the wild type in this study | C-type | C-type |
| CL1 | 1^st^ start codon→nonsense | D-type | C-type |
| C33 | N-terminal deletion | D-type | D-type |
| CH | N-terminal sequence exchange | H-type | H-type |
| CE | N-terminal sequence exchange | E-type | E-type |
| D | No, defined as the wild type in this study | D-type | D-type |
| DC | N-terminal extension | C-type | C-type |
| DCL1 | N-terminal extension+1^st^ start codon→nonsense | D-type | C-type |
| DH | N-terminal extension | H-type | H-type |
| DE | N-terminal extension | E-type | E-type |

**Supplementary Table S2. The primer sequences used in plasmid construction** **in this study**

| **Primer** | **Sequence (5’-3’)** |
| --- | --- |
| **Forward** |  |
| P1 | aaacgaattcggatcttccagagattttttcacctctgcctaatca |
| P5 | ctcgtggtggacttctctca |
| P9 | gactcactatagggcgaattcatcctgccttgatgcctttatatg |
| P10 | gcaactttttcacctctgcctaatcatctcatg |
| P14 | gactcactatagggcgaattcatcctgcgttaatgcccttg |
| P15 | cacctctgcctaatcatctcttgttcatgtcctactgttcaa |
| P17 | acaagagctacagcacgggaggttggtcttccaaacctc |
| P20 | tacagcatggggacgaatctttctgttcc |
| P22 | tctcaacggcgagaaggggcatggggcagaatctttctgttcccaat |
| P24 | tggacggtccctctcgaatgggggaagaatctttctgttccca |
| P26 | ggtcttccaaacctcgacaaggcatggggacgaatctttccaccagcaat |
| P30 | acctctctcaacggcgagaaggggcatggggcagaatctt |
| P32 | tggacggtccctctcgaatgggggaagaatctttccaccagcaatc |
| P34 | aggtctatataagcagagctctctggctaactgcacctgcaactttttc |
| P35 | agactcgtggtggacttc |
| P39 | gggagacccaagctggctagcatgggaggttggtcttccaaa |
| P41 | gtcatcctcaggccctgcagtggaactcc |
| P42 | gaccctgcaccgaacctggagagcacaacat |
| P45 | gggagacccaagctggctagcatggggacgaatctttctgttcc |
| P46 | gggagacccaagctggctagcatgggagcacctctctcaacggcgaga |
| P47 | cctctctcaacggcgagaaggggcatggggcagaatctttctgttcccaa |
| P48 | gggagacccaagctggctagcatggggctttcttggacggtccctc |
| P49 | tttcttggacggtccctctcgaatgggggaagaatctttctgttcccaa |
| P50 | atttctctatcgataggtaccgaccaccgtgaacgccca |
| P53 | atttctctatcgataggtacccctgaacatgcagttaatcattacttc |
| P55 | atttctctatcgataggtacccaccatattcttgggaacaagagc |
| P75 | tgcatgatgtggtattgggggc |
| **Reverse** |  |
| P2 | gcgaattcaactgccgttcgacgataaaaagttgcatggtgctgg |
| P6 | gagatgattaggcagaggtgaaaaagttgcatggtgctggt |
| P11 | ggcagaggtgaaaaagttgcatggtgctggtga |
| P12 | gaagtccaccacgagtctagactctgtggtattgtgaggattcttg |
| P13 | cactatagaatactcaagcttccgatacagagctgaggcgg |
| P16 | gaagtccaccacgagtctagactctgcggtattgtgaggattcttg |
| P18 | tcccgtgctgtagctcttgttccc |
| P19 | gaagtccaccacgagtctagactctgtggtattgtgaggattcttg |
| P21 | agattcgtccccatgctgtagctcttgttcccaagaa |
| P23 | ttctcgccgttgagagaggtgctcccatgctgtagctctt |
| P25 | gagagggaccgtccaagaaagccccatgctgtagctcttg |
| P27 | gaggtttggaagaccaacctcccatgctgtagatcttgttccc |

**Supplementary Table S2 (continued). The primer sequences used in plasmid construction** **in this study**

| **Primer** | **Sequence (5’-3’)** |
| --- | --- |
| **Reverse** |  |
| P28 | tggtgaaaggttgtggaattccactgcatggcctgaggatg |
| P29 | gaggtttggaagaccaacctcccgtgctgtagatcttgttcc |
| P31 | ttctcgccgttgagagaggtgctcccatgctgtagatcttg |
| P33 | gagagggaccgtccaagaaagccccatgctgtagatctt |
| P36 | ggtttaaacgggccctctagagatctcgaatagaaggaaagaagtca |
| P37 | tgagagaagtccaccacgag |
| P38 | ggtttaaacgggccctctagagatctcgtactgaaggaa |
| P40 | ggtttaaacgggccctctagattaaatgtatacccaaagacaaaagaaa |
| P43 | ggagttccactgcagggcctgaggatgac |
| P44 | atgttgtgctctccaggttcggtgcagggtc |
| P51 | tgtttttggcgtcttccatggttagatgattaggcagaggtgaaaaa |
| P52 | tgtttttggcgtcttccatggaccaatttatgcctacagcctcc |
| P54 | cagtaccggaatgccaagcttgctgtagctcttgttcccaagaa |
| P56 | cagtaccggaatgccaagctttgttgtggagttccactgcatg |
| P74 | ccccaataccacatcatgcatataactgaaagccaaacagtgg |

**Supplementary Table S3. The recombinant plasmids and their construction procedures in this study**

| **Plasmid** | **Vector** | **Construction procedure**  **(①: PCR；②: overlap PCR; ③: seamless cloning;**  **④: site-directed mutation)** |
| --- | --- | --- |
| ***1.0×HBV genome amplified from HepG2.2.15 supernatant*** | | |
| 1.0D | EcoRV linearizd pCE-Zero | ①: F1 (P1+P2)^#^_;_ ③: F1+vector |
| ***1.3×HBV genome, FL (1043-3215, 1-2017) in GTC,***  ***FL (1043-3182, 1-2017) in GTD*** | | |
| C | EcoRI and HindIII linearized pGEM-3Z | ①: F1 (P9+P11); F2 (P10+P12); F3 (P5+P11); F4 (P10+P13);  ②: F1+F2→F12; F3+F4→F34;  ③: F12+F34+vector |
| D |  | ①: F1 (P14+P6); F2 (P15+P16); F3 (P5+P6); F4 (P15+P13);  ②: F1+F2→F12; F3+F4→F34;  ③: F12+F34+vector |
| ***1.3×HBV genome harboring mutation in preS1 of LHBs*** | | |
| CL1 | EcoRI and XbaI linearized C | ①: F1 (P9+P18); F2 (P17+P19); ③: F1+F2+vector |
| C33 |  | ①: F1 (P9+P21); F2 (P20+P19); ③: F1+F2+vector |
| CH |  | ①: F1 (P9+P23); F2 (P22+P19); ③: F1+F2+vector |
| CE |  | ①: F1 (P9+P25); F2 (P24+P19); ③: F1+F2+vector |
| DC | EcoRI linearized D | ①: F1 (P14+P27); F2 (P26+P28); ③: F1+F2+vector |
| DCL1 |  | ①: F1 (P14+P29); F2 (P26+P28); ③: F1+F2+vector |
| DH |  | ①: F1 (P14+P31); F2 (P30+P28); ③: F1+F2+vector |
| DE |  | ①: F1 (P14+P33); F2 (P32+P28); ③: F1+F2+vector |
| ***1.05×HBV genome under the control of CMV promoter,***  ***FL (1809-3215, 1-1988) in GTC or FL (1809-3182, 1-1988) in GTD*** | | |
| PC-C  PC-CL1  PC-C33  PC-CH  PC-CE | SacI and XbaI linearized pcDNA3.1 | ①: F1 (P34+P19); F2 (P35+P36); ③: F1+F2+vector |
| PC-D  PC-DCL1  PC-DC  PC-DH  PC-DE |  | ①: F1 (P34+P37); F2 (P5+P38); ③: F1+F2+vector |
| ***LMS or LHBs expression*** | | |
| PC-LMS | NheI and XbaI linearized pcDNA3.1 | ①: F1 (P39+P40); ③: F1+vector |
| C-L |  | ④: Mutate start codon (ATG) of preS2 with P41 and P43 into CTG; Mutate start codon (ATG) of S with P42 and P44 into CTG |
| C33-L |  | ①: F1 (P45+P40) from C-L; ③: F1+vector |
| CH-L |  | ①: F1 (P47+P40) from C-L; F2 (P46+P40) from F1; ③: F2+vector |
| CE-L |  | ①: F1 (P49+P40) from C-L; F2 (P48+P40) from F1; ③: F2+vector |
| ***Luciferase reporter plasmids of HBV promoters*** | | |
| CP-pg | KpnI and NcoI linearized pGL3-basic | ①: F1 (P50+P51); ③: F1+vector |
| CP-preC |  | ①: F1 (P50+P52); ③: F1+vector |
| SPI | KpnI and HindIII linearized pGL3-basic | ①: F1 (P53+P54); ③: F1+vector |
| SPII |  | ①: F1 (P55+P56); ③: F1+vector |

^#^The primers used for construction of the plasmids are showed in the parentheses. Their sequences could be found in Supplementary table S2. CMV, cytomegalovirus; CP, core promoter; F, fragment; FL, HBV genome full length; GTC, genotype C; GTD, genotype D; LMS, LHBs/MHBs/SHBs; LHBs, HBV large surface protein; Pol, polymerase; P1, primer No. 1.

**Supplementary Table S4 A brief summary of the impacts of mutants’ LHBs on activity of HBV promoters in comparison with that of the mock**

| LHBs type | CP-preC | CP-pg | SPI | SPII |
| --- | --- | --- | --- | --- |
| C-L | ↓↓↓ | — | ↑ | — |
| C33-L | — | — | ↑ | — |
| CH-L | ↓↓↓ | — | ↑↑ | ↑ |
| CE-L | ↓ | ↑ | ↑ | — |

↑, significantly higher; ↓, significantly lower; —, no significant difference; 1 to 3 arrows represent *p* values less than 0.05, 0.01 and 0.001, respectively. HBV, hepatitis B virus; LHBs, HBV large surface protein; CP, core promoter; SP, surface promoter.

**Supplementary Table S5 The replication characteristics of HBV mutants in comparison with that of wild type strain^#^**

| Strain | LHBs-N-terminus | Pol-spacer | Extracellular  HBsAg | Intracellular  HBsAg | Coefficient of  HBsAg secretion | Extracellular  LHBs^*^ | Intracellular  LHBs^*^ | Extracellular  SHBs^*^ | Intracellular  SHBs^*^ | HBeAg | Intracellular HBc^*^ | Extracellular  HBV DNA | Replicative  HBV DNA^*^ | Infectivity |
| --- | --- | --- | --- | --- | --- | --- | --- | --- | --- | --- | --- | --- | --- | --- |
| CL1 | D-type | C-type | — | — | ↑ | ↑ | ↓ | — | ↓ | — | — | ↑ | — | ↑↑↑ |
| C33 | D-type | D-type | ↑ | — | ↑↑ | ↑ | ↓ | — | ↓ | ↑ | ↓ | ↑↑ | ↑ | ↑↑ |
| CH | H-type | H-type | ↓↓↓ | ↓↓ | ↓↓ | ↓ | — | ↓ | ↓ | — | ↓ | ↑ | ↑ | ↓ |
| CE | E-type | E-type | ↑↑ | — | ↑↑ | — | ↓ | — | ↓ | — | — | — | ↑ | ↑↑ |
| DCL1 | D-type | C-type | ↓ | — | — | — | ↑ | — | ↑ | ↑↑ | — | ↓↓ | ↓ | ↑↑ |
| DC | C-type | C-type | ↓↓↓ | — | ↓↓ | ↓ | ↑ | ↓ | ↑ | ↑↑ | — | ↓↓↓ | ↓ | ↓↓↓ |
| DH | H-type | H-type | ↓↓↓ | — | ↓↓ | ↓ | ↑ | ↓ | ↑ | ↓ | ↑ | — | — | ↓↓↓ |
| DE | E-type | E-type | ↓ | — | — | — | — | — | ↑ | — | ↑ | ↑ | ↓ | ↓ |

^#^C-derived and D-derived mutants were compared with the corresponding wild type C and D strains, respectively; ↑, significantly higher than C or D; ↓, significantly lower than C or D; —, no significant difference with C or D; 1 to 3 arrows represent *p* values less than 0.05, 0.01 and 0.001, respectively. *evaluation by empirical observation rather than statistical analyses. HBV, hepatitis B virus; HBsAg, hepatitis B surface antigen; LHBs, HBV large surface protein; SHBs, HBV small surface protein; HBc, HBV core protein.

**
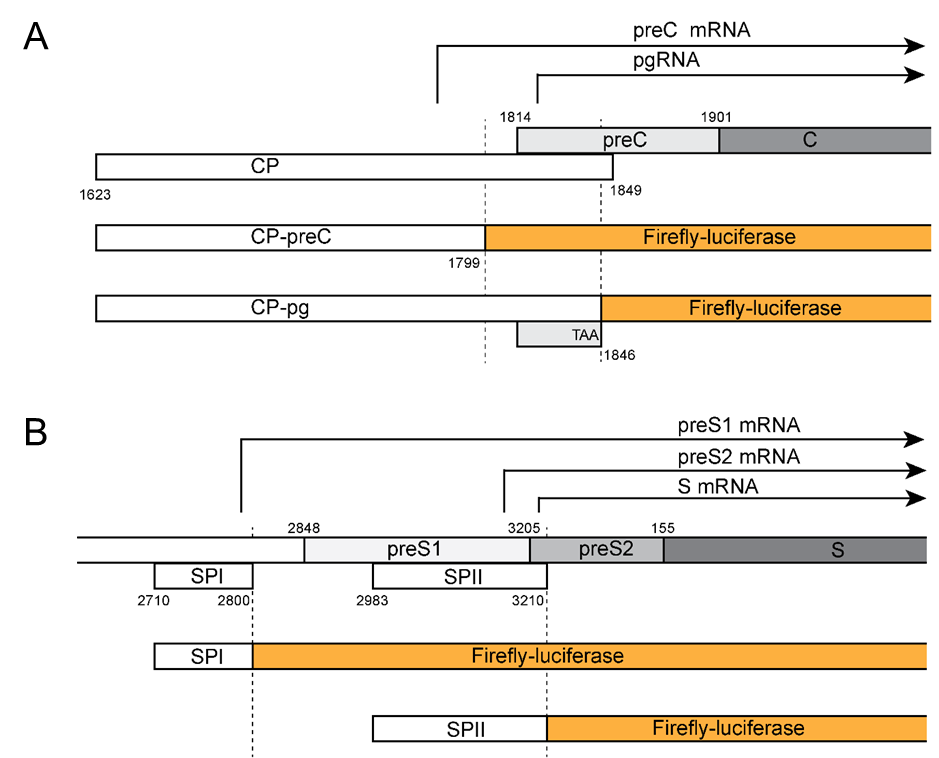
**

**Supplementary Figure S1**. Schematic representation of HBV promoters for dual luciferase reporter assay. HBV promoters are represented by open boxes. The preC/C and preS/S ORFs are indicated by gray boxes. Transcriptional start sites of preC, C, preS1, preS2 and S mRNA are indicated by arrows. **(A)** The activities of CP-preC and CP-pg reflect transcriptional levels of CP in preC mRNA and pgRNA, respectively. **(B)** The activities of SPI and SPII reflect transcriptional levels of preS1 and preS2/S mRNA, respectively. CP, core promoter; preC, precore; C, core; pgRNA, pregenomic RNA; preS, presurface; S, surface; SP, surface promoter.


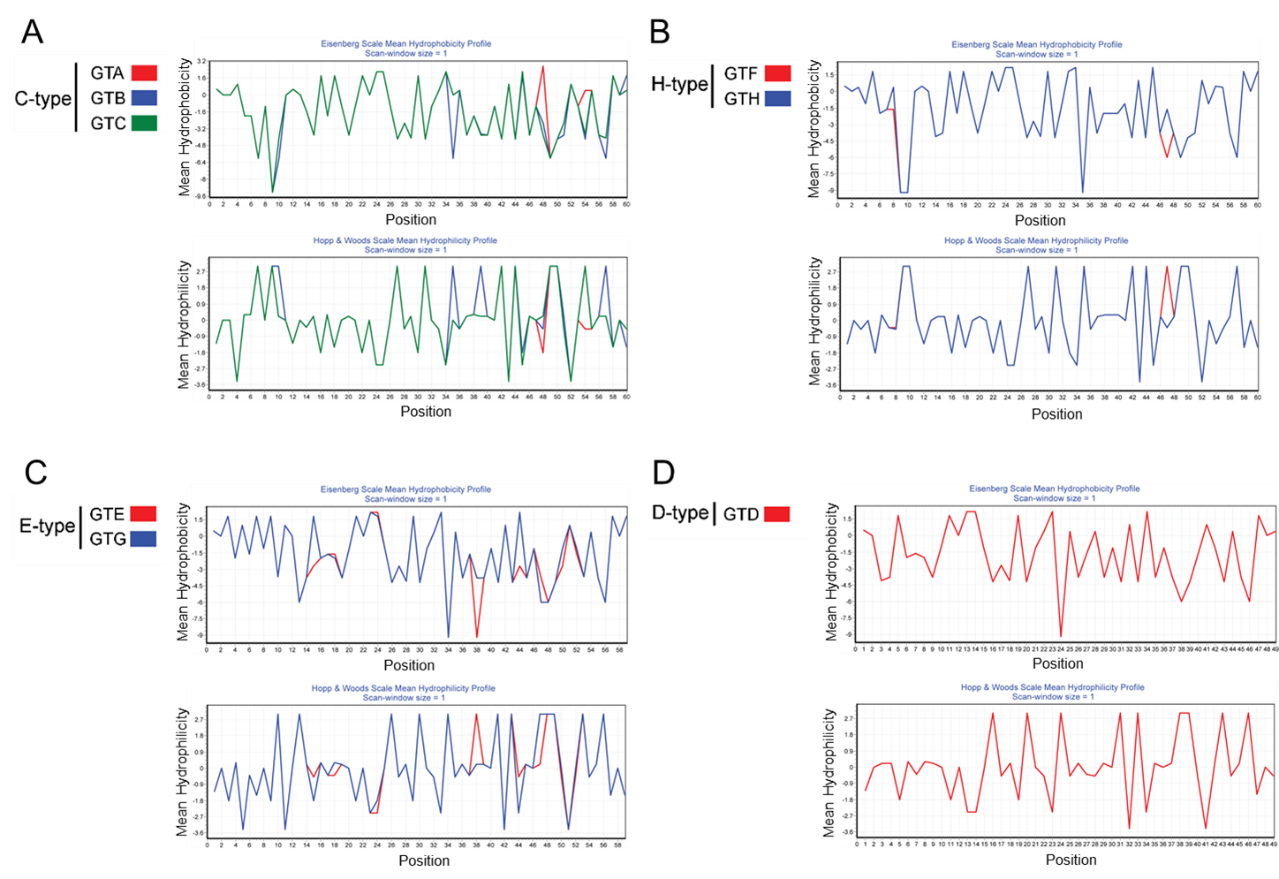


**Supplementary Figure S2**. Comparison of hydrophobicity and hydrophilicity profiles of preS1 N-termini. Eisenberg scale mean hydrophobicity profiles **(upper panel in A,B,C,D)** or Hopp & Woods scale mean hydrophilicity profiles **(lower panel in A,B,C,D)** are generated based on preS1 N-terminal amino acid consensuses from GTA to GTH by BioEdit software. GT, genotype.


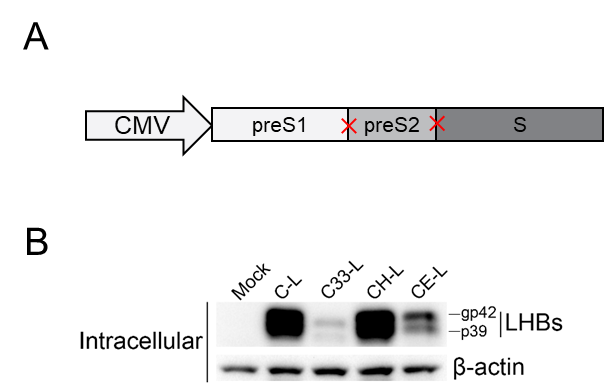


**Supplementary Figure S3**. Construction and expression of LHBs. **(A)** preS/S gene of C-derived strains were cloned into pcDNA3.1 and the plasmids expressing LHBs alone (C-L, C33-L, CH-L and CE-L) were obtained by mutating start codon ATG of SHBs and MHBs into codon CTG. **(B)** Three days post transfection of LHBs plasmids in HepG2 cells, intracellular LHBs were detected by Western blot. The glycosylated (gp) and nonglycosylated (p) forms of LHBs are indicated. CMV, cytomegarovirus; LHBs, HBV large surface protein; preS, presurface; S, surface.


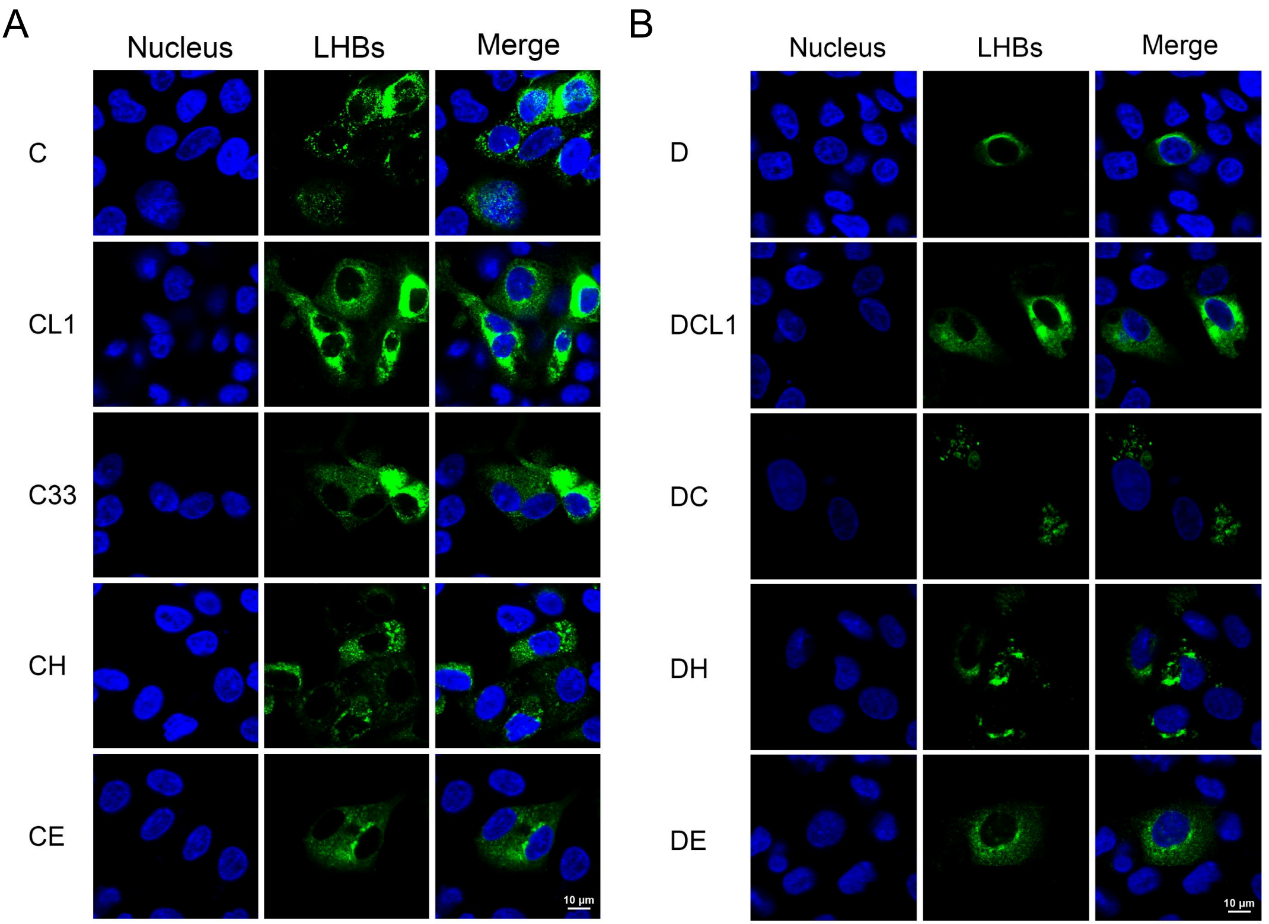


**Supplementary Figure S4**. Impact of preS1 N terminal sequences on subcellular distribution of LHBs. HepG2 cells were transfected with plasmids containing 1.3-mer HBV genome from C-derived (A) or D-derived (B) strains for three days. The mouse monoclonal anti-preS1 antibody (Santa Cruz Biotechnology) was used for LHBs detection, and nuclei were stained by DAPI. Scale bar represents 10 μm. LHBs, HBV large surface protein.


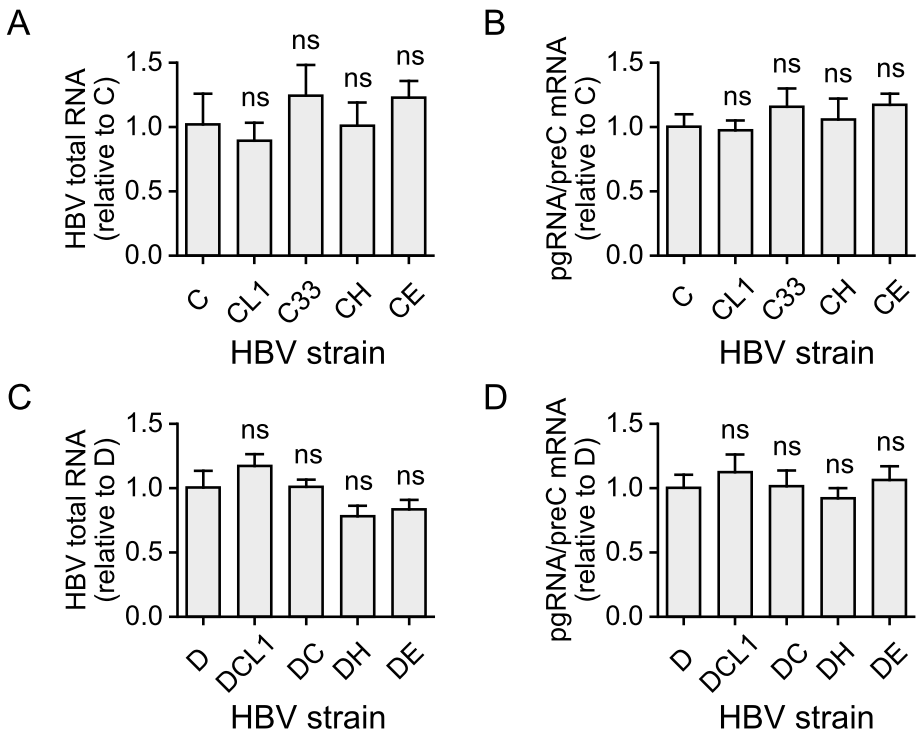


**Supplementary Figure S5**. Impact of preS1 N terminal sequences on intracellular HBV total RNA and pgRNA/preC mRNA post transfection. Plasmids containing 1.3-mer HBV genome from C-derived (A,B) and D-derived (C,D) strains were transfected into HepG2 cells. Total RNA was extracted from cultured cells at day 3 post transfection. Reverse transcription-quantitative PCR was used to detect HBV total RNA (A,C) and pgRNA/preC mRNA (B,D). Ribosomal protein S11 gene was used as endogenous control. The histograms show mean values from one representative experiment; Bars indicate Standard Deviation. The *p* values were determined using Student’s t test; ns represents no significant.


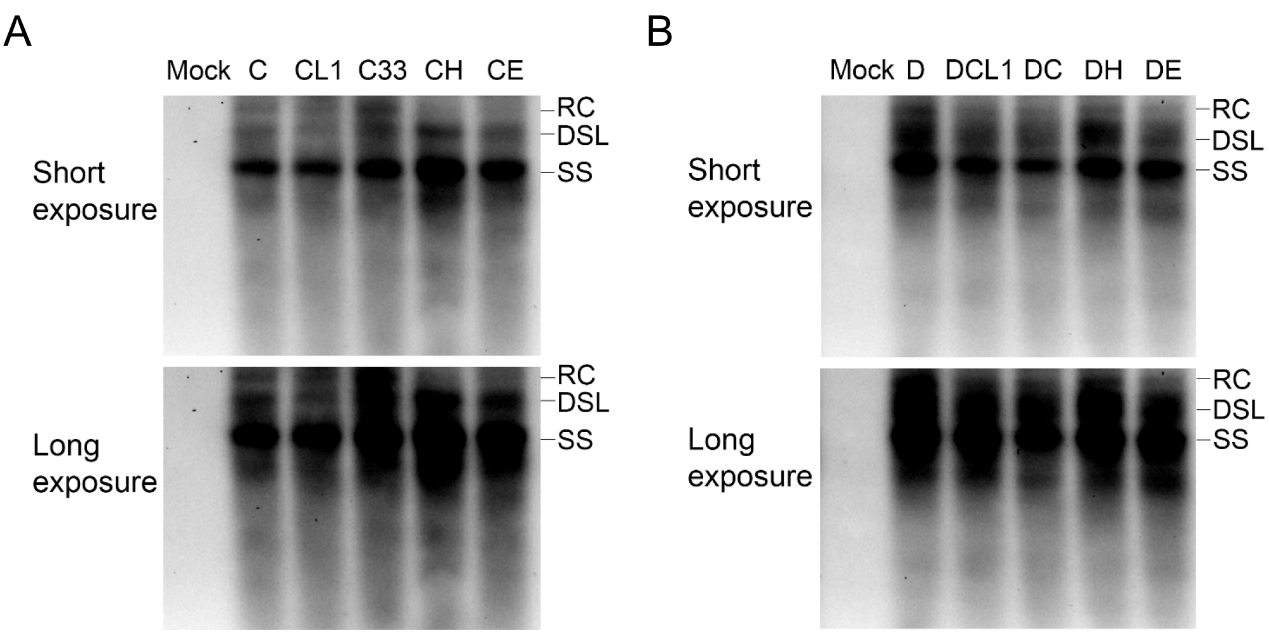


**Supplementary Figure S6**. Impact of preS1 N-terminal sequences on intracellular replicative HBV DNA. HepG2 cells were transfected with plasmids containing 1.3-mer HBV genomes from C-derived (A) or D-derived (B) strains. RC, relaxed circular; DSL, double-stranded linear; SS, single stranded.


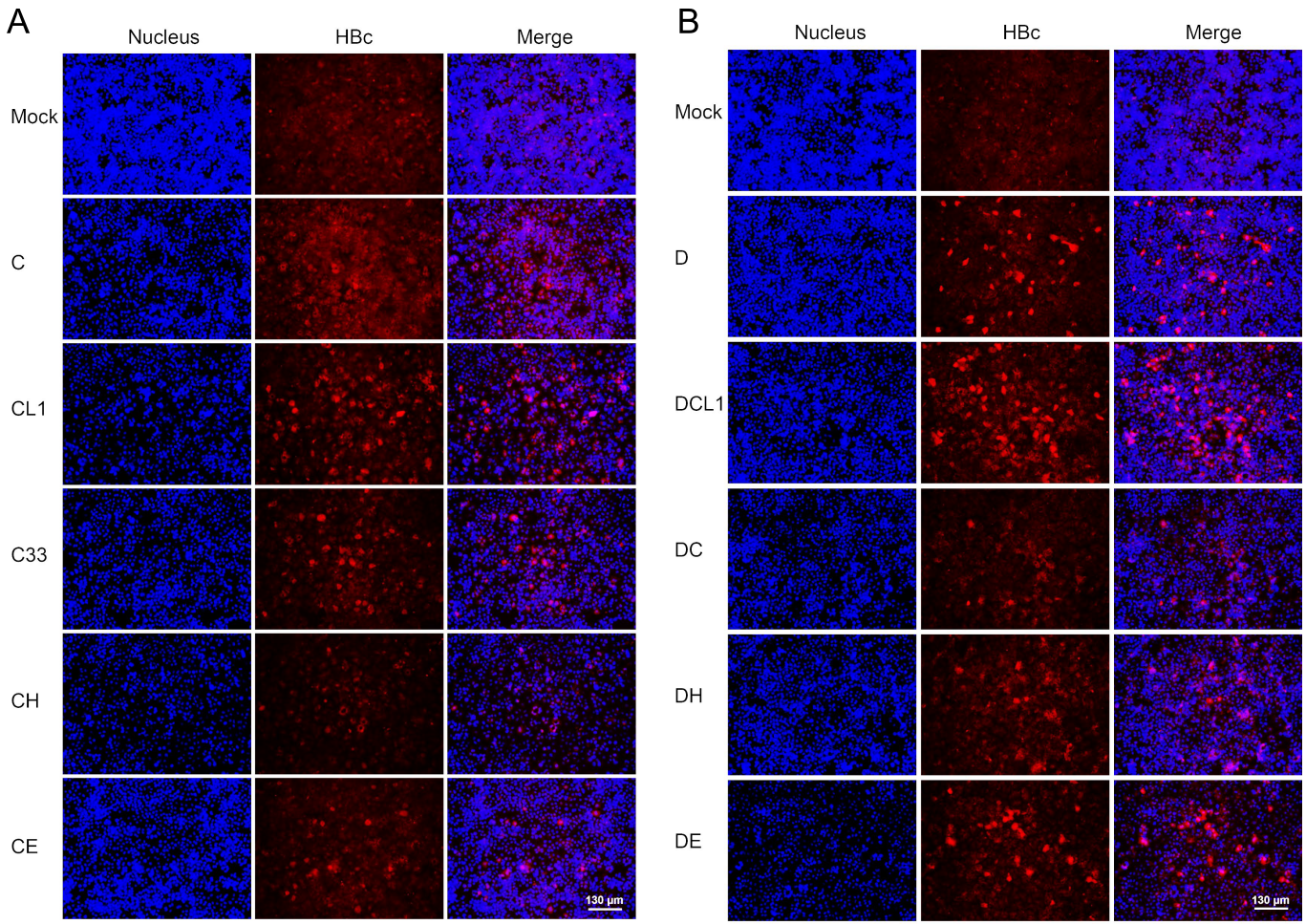


**Supplementary Figure S7**. Impact of preS1 N-terminal sequences on HBV infectivity. HepG2-NTCP cells were infected with HBV preparations from C-derived (A) or D-derived strains (B). The multiplicity of infection of 5000 genome equivalents per cell were inoculated into HepG2-NTCP cells. Cells were stained with anti-HBc antibody (Fitzgerald Industries International) and nuclei were stained by DAPI at day 7 post infection. Scale bar represents 130 μm. HBc, HBV core protein.


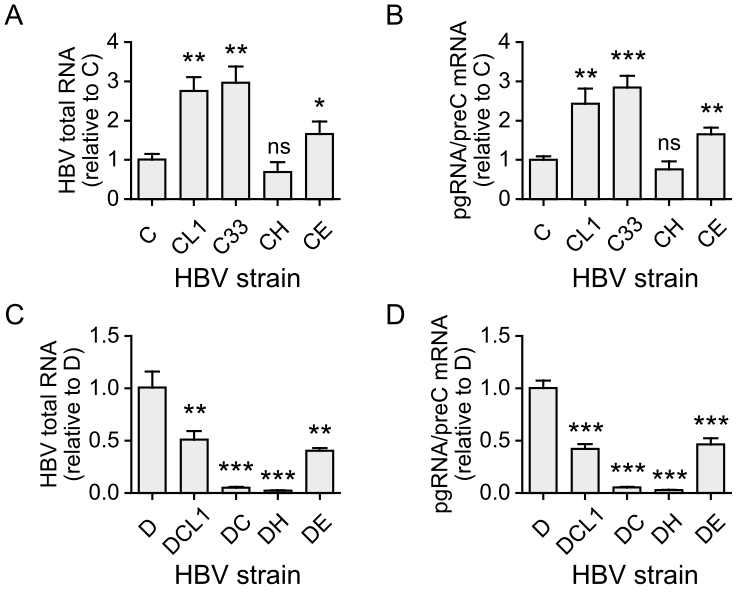


**Supplementary Figure S8**. Impact of preS1 N terminal sequences on intracellular HBV total RNA and pgRNA/preC mRNA post HBV infection. HepG2-NTCP cells were infected with HBV preparations from C-derived (A,B) or D-derived strains (C,D). The multiplicity of infection of 5000 genome equivalents per cell were inoculated into HepG2-NTCP cells. Total RNA was extracted from cultured cells at day 7 post infection. Reverse transcription-quantitative PCR was used to detect HBV total RNA (A,C) and pgRNA/preC mRNA (B,D). Ribosomal protein S11 gene was used as endogenous control. The histograms show mean values from one representative experiment; Bars indicate Standard Deviation. The *p* values were determined using Student’s t test; ns represents no significant, **p*<0.05, ***p*<0.01, ****p*<0.001.
